# Long-Chain Omega-3 Polyunsaturated Fatty Acids May Be Beneficial for Reducing Obesity—A Review

**DOI:** 10.3390/nu2121212

**Published:** 2010-12-09

**Authors:** Jonathan D. Buckley, Peter R. C. Howe

**Affiliations:** 1 Nutritional Physiology Research Centre, University of South Australia Adelaide, South Australia, 5000, Australia; Email: peter.howe@unisa.edu.au; 2 Sansom Institute for Health Research, University of South Australia Adelaide, South Australia, 5000, Australia

**Keywords:** weight loss, body fat, gene expression, satiety, lean tissue anabolism

## Abstract

Current recommendations for counteracting obesity advocate the consumption of a healthy diet and participation in regular physical activity, but many individuals have difficulty complying with these recommendations. Studies in rodents and humans have indicated that long-chain omega-3 polyunsaturated fatty acids (LC n-3 PUFA) potentially elicit a number of effects which might be useful for reducing obesity, including suppression of appetite, improvements in circulation which might facilitate nutrient delivery to skeletal muscle and changes in gene expression which shift metabolism toward increased accretion of lean tissue, enhanced fat oxidation and energy expenditure and reduced fat deposition. While LC n-3 PUFA supplementation has been shown to reduce obesity in rodents, evidence in humans is limited. Epidemiological associations between LC n-3 PUFA intakes and obesity are inconclusive but small cross-sectional studies have demonstrated inverse relationships between markers of LC n-3 PUFA status and markers of obesity. Human intervention trials indicate potential benefits of LC n-3 PUFA supplementation, especially when combined with energy-restricted diets or exercise, but more well-controlled and long-term trials are needed to confirm these effects and identify mechanisms of action.

## 1. Introduction

Obesity is increasing in epidemic proportions throughout the developed and developing world [1,2] bringing with it increased morbidity and mortality [3,4]. If we are to avoid prohibitive increases in health costs, and adverse health consequences for an increasing proportion of society, feasible and affordable strategies to combat the rising prevalence of obesity must be found.

Current recommendations from most public health bodies for reducing body fat are based on increasing physical activity [5] and eating a healthy balanced diet [6]. However, many people have difficulty complying with these lifestyle changes, particularly over the longer term. Indeed, a recent meta-analysis showed that almost half of any initial weight loss achieved through diet or exercise is regained after one year [7]. Hence, despite widespread recommendations from public health bodies to improve diet and physical activity habits the prevalence of obesity continues to rise. Accordingly, more acceptable and sustainable strategies for reducing body fat must be found.

Considerable efforts have been directed to investigating the benefits of the long-chain omega-3 polyunsaturated fatty acids (LC n-3 PUFA), notably eicosapentaenoic acid (EPA) and docosahexaenoic acid (DHA), for improving cardiovascular health and reducing cardiovascular disease (CVD) risk. However, despite the current obesity epidemic and a growing body of evidence indicating that the regular consumption of these fatty acids can influence fat metabolism, little attention has been paid to the potential for EPA and DHA to reduce obesity.

This review extends the work described in a previous review by these authors [8], providing a more detailed examination of the included studies and introducing the potential effects of LC n-3 PUFA on lean tissue accretion as a mechanism for obesity reduction.

## 2. LC n-3 PUFA and Body Fat Reduction in Animals

### 2.1. Protection against Body Fat Gain

A growing number of studies have shown that incorporating LC n-3 PUFA into high-fat obesogenic diets fed to rodents reduces body fat accumulation [9,10,11,12,13], although one study in diabetic rats reported that supplementing a high-fat diet with LC n-3 PUFA exacerbated, rather than reduced, weight gain [14]. 

Cunnane *et al.* [11] fed 6-week old male *ln*/*ln* and *ob*/*ob* C57BL/6J mice synthetic diets that were supplemented with 10% (w/w) evening primrose oil (predominantly omega-6 (n-6) PUFA) or 10% cod liver oil (predominantly LC n-3 PUFA) for 16 weeks. Overall, the *ob*/*ob* mice gained significantly more weight than the *ln*/*ln* mice irrespective of the diet consumed. The *ob*/*ob* mice that consumed the cod liver oil finished the study with a significantly lower body weight than the *ob*/*ob* mice that consumed the evening primrose oil, but this was due in part to a slight difference in starting weights as the tendency for less weight gain in the animals fed the cod liver oil did not reach statistical significance compared with evening primrose oil. The difference in body weight between treatment groups was not related to differences in energy intake, but was instead ascribed to differences in incorporation of LC n-3 and n-6 PUFA into liver, brown adipose tissue and white adipose tissue. The *ob*/*ob* mice fed the cod liver oil demonstrated greater incorporation of LC n-3 PUFA and reduced incorporation of n-6 PUFA into these tissues which has the potential to influence gene expression that would alter their metabolic activity leading to reductions in body fat accumulation [15].

Hainault *et al.* [12] subsequently fed six week old male Wistar rats one of three high-fat diets (50% of energy) or a low fat control diet (10% corn oil, 20% casein, 70% starch) *ad libitum* for 16–20 days. The high fat diets consisted of 48% lard, 2% corn oil (high-lard diet), 35% lard, 15% corn oil (lard–corn oil diet) or 33% lard, 2% corn oil and 15% fish oil (lard-fish oil diet), together with 20% casein and 30% starch. Food intake and body weights were similar for all diets, but rats consuming the lard–fish oil diet had 20% and 30% less subcutaneous (inguinal) and visceral (retroperitoneal and epididymal) adipose tissue respectively by the end of the study period, suggesting that the fish oil had provided a protective benefit against the accumulation of body fat. 

Belzung *et al.* [10] demonstrated that this protective effect of fish oil exhibited a dose-response relationship. They fed 7-week old male Wistar rats a control diet (standard chow) or one of three high–fat diets (20% fat w/w) containing high, medium and low LC n-3 PUFA content (40.6%, 18.6% and 0.7% of fatty acids, respectively) for four weeks. The three high fat diets resulted in greater body fat accumulation compared with the control diet, with no differences in increases in body weight, subcutaneous fat or mesenteric fat. However, the accumulation of epididymal fat was less in the high LC n-3 PUFA group and there was a dose dependent attenuation of the increase in retroperitoneal fat mass. These differences in epididymal and retroperitoneal fat were due to reduced adipocyte hypertrophy rather than to any reduction in adipocyte number. The authors proposed that the reductions in fat were mediated by a lowering of circulating triglycerides given that plasma triglyceride concentrations were lower with the medium and high LC n-3 PUFA treatments, and the plasma triglyceride concentrations correlated positively with retroperitoneal and epididymal adipocyte size. The reduction in adipocyte hypertrophy was consistent with the findings of an earlier study by Parrish *et al.* [16]. Parrish and co-workers fed male Wistar Rats high-fat diets containing 20% triglycerides from fish oil or 20% triglycerides from lard for three weeks and found no difference in body weight, but significantly less perirenal and epididymal fat in the fish oil fed group. This effect was the result of a reduction in adipocyte size with no difference in adipocyte number. Thus, it appears that the protective benefits of LC n-3 PUFA against the accumulation of body fat may be achieved through a lesser accumulation of fat in existing adipocytes, with these effects being primarily evident in visceral fat depots and exhibiting a dose-response relationship.

The preferential reduction in the accumulation of visceral fat has been confirmed in two subsequent studies [9,13] and may be mediated primarily by DHA [13]. Baillie *et al.* [9] fed two groups of 3–4 week old male Fisher 344 rats a high fat diet containing 40% of energy as either fish oil or corn oil for six weeks. Epididymal fat pads from rats fed the fish oil diet weighed 25% less than those of the rats fed corn oil by the end of the six week study period. Ruzickova *et al.* [13] subsequently fed 3–4 month old male C57BL/6 mice a low-fat control diet (3.4% fat (w/w)), one of two high-fat control diets (20.0% and 35.2% fat (w/w)), or one of seven experimental diets which consisted of the high-fat control diets admixed with 15% or 45% (w/w) EPA or DHA for five weeks. By the end of the study period it was found that EPA and DHA attenuated the accumulation of epididymal fat, but had a limited effect on the accumulation of subcutaneous fat. Importantly, it was further determined that this effect was promoted by a low ratio of EPA/DHA, suggesting that DHA, rather than EPA, was primarily responsible for promoting the anti-obesity effect of an increased LC n-3 PUFA intake.

Therefore, there are a number of studies in rodents which indicate that the dietary intake of LC n–3 PUFA can attenuate increases in diet-induced obesity, particularly increases in visceral (epididymal fat). However, a recent study [14] which compared feeding different high-fat diets for six weeks on the development of obesity in male C57BL/KsJ-lepr^db^/lepr^db^ mice with diabetes found that feeding a high–fat diet (30% of energy) containing LC n-3 PUFA exacerbated weight gain compared with the same diet containing safflower oil (rich in n-6 fatty acids). This exacerbation of weight gain only occurred in mice with diabetes; in non-diabetic mice the safflower diet resulted in increased weight gain compared with a low-fat control diet but the LC n-3 PUFA diet did not result in any greater weight gain. As body fat was not measured, it is unclear whether the differences in weight gain between diets were due to differences in body fat deposition. Nevertheless, the increased weight gain in the mice with diabetes fed the LC n-3 PUFA diet suggests that these fatty acids may exert quite different metabolic effects in diabetes, at least in mice.

### 2.2. LC n-3 PUFA for Reducing Existing Obesity

While a number of studies in rodents have shown that the incorporation of LC n-3 PUFA into an obesogenic diet can attenuate the accumulation of body fat, at least in non-diabetic animals, there have been limited studies which have examined whether incorporating LC n-3 PUFA into a diet can reduce established adiposity. In the only study to specifically examine this question, Huang *et al.* [17] demonstrated that the intake of LC n-3 PUFA could reduce body fat in mice that had been made obese by prior feeding of a high-fat diet. In this study, male C57B1/l mice were fed a high fat (59% of energy) or low-fat (10% of energy) control diet for 13 weeks. The mice were then classified as diet–induced obese or diet-resistant according to whether they fell within the upper or lower tertiles of body weight gain. The diet-induced obese mice were then subjected to a six week dietary intervention which consisted of feeding either the original high fat diet, a high fat diet with a high LC n-3 PUFA content (59% of energy from fish oil), or the low-fat control diet. The LC n-3 PUFA and low-fat diets reduced body weight to the same level as the diet resistant mice and the controls that had been maintained on the low-fat diet, indicating that both LC n-3 PUFA and a low-fat diet were effective for weight loss. The loss of body weight after the mice were switched to the LC n-3 PUFA diet could not be attributed to a reduction in food intake. However, the mice that were switched to the LC n-3 PUFA diet exhibited the lowest food efficiencies (*i.e.*, lowest weight gain per unit energy intake) indicating that the LC n-3 PUFA reduced metabolic efficiency.

Therefore, it appears that, in rodent models, the dietary intake of LC n-3 PUFA may be effective for retarding body weight and body fat gain during the consumption of an obesogenic diet, and for reducing body weight in animals that are already obese. However, there is evidence, albeit somewhat limited, that the dietary intake of LC n-3 PUFA may exert different metabolic effects in diabetic animals, resulting in an exacerbation of weight gain when a high-fat obesogenic diet is consumed.

## 3. LC n-3 PUFA and Body Fat Reduction in Humans

While there is evidence from a considerable number of animal studies that LC n-3 PUFA can reduce body fat, their impact on body composition in humans is less certain due to a lack of appropriately designed studies of adequate size and duration. Furthermore, despite dietary energy restriction and/or exercise being the cornerstones of treatment for obesity, few studies have investigated the efficacy of LC n-3 PUFA for reducing body fat when combined with these treatments.

### 3.1. Observational Studies—Relationships between LC n-3 PUFA Intake and Body Weight

A number of large long-term prospective cohort studies have examined the effects of LC n-3 PUFA supplementation on cardiovascular health but none have specifically examined effects on body weight or body fat. Nevertheless, the Health Professional Follow-Up Study, a prospective cohort study with 12 years of follow-up which examined the effects of LC n-3 PUFA intake on risk of stroke in 43,671 men [18], reported that, at baseline, men with high fish consumption (consuming fish ≥5 times per week) were less likely to be overweight than those with low fish consumption (consuming fish less than once per month), and the percentage of overweight volunteers was inversely related to the LC n-3 PUFA intake. Conversely, in the Nurses’ Health Study [19], which was a prospective cohort study in 79,839 women with a 14 year follow-up which examined the relationship between the intake of fish and LC n-3 PUFA and the risk of stroke, higher intakes of fish and LC n-3 PUFA were associated with a higher prevalence of obesity, with the proportion of participants with a body mass index (BMI) ≥ 29 kg/m^2^ increasing progressively as fish intake increased from less than once per month to ≥5 times per week. While the higher prevalence of obesity associated with fish intake could be accounted for by a higher energy intake, this was not the case for LC n-3 PUFA intake.

Thus, the limited evidence available from large prospective cohort studies which have assessed the potential relationships between LC n-3 PUFA intake and body weight is conflicting. It is not clear whether the different findings of the two studies that have investigated this issue are due to confounding or to gender differences in the effect of LC n-3 PUFA on body fat. This raises the possibility that the effect of LC n-3 PUFA on reducing body weight and/or body fat in humans may be limited to males.

A limitation of the large epidemiological studies is that dietary intakes are assessed using semi–quantitative food frequency questionnaires which might not be particularly sensitive in terms of their ability to accurately quantify intakes of particular fatty acids. A different approach is to use tissue content of LC n-3 PUFA as a biomarker of intake. Garaulet *et al.* [20] examined the fatty acid composition of adipose tissue samples of 84 obese patients (BMI 27–35 kg/m^2^) and found that abdominal obesity as assessed by waist-hip ratio and visceral abdominal fat area (by computed tomography) were inversely related to the LC n-3 PUFA content (in particular the DHA content) of perivisceral and omental adipose tissue samples. In a subsequent study, the same group showed that the LC n-3 PUFA content of the subcutaneous adipose tissue (but not other adipose tissue depots) was inversely related to adipocyte size [21]. Taken together, these data indicate that a higher LC n-3 PUFA status is associated with reduced abdominal obesity as a result of a reduction in adipocyte size. Similarly, in a more recent study, Micaleff *et al.* [22] measured plasma LC n-3 PUFA concentrations as a biomarker of dietary LC n-3 PUFA intake in 124 healthy adults (BMI 20–40 kg/m^2^) and reported that the plasma LC n-3 PUFA levels (% of total plasma fatty acids) were inversely related to BMI, waist circumference and hip circumference, again suggesting that a higher plasma LC n-3 PUFA status might protect against obesity. 

### 3.2. Randomized Controlled Trials

While cross-sectional studies are useful for identifying associations between LC n-3 PUFA intake and obesity, they cannot demonstrate a cause and effect relationship since people who consume a high intake of LC n-3 PUFA might also undertake other diet and/or lifestyle practices which could account for lower body fat. In order to directly assess the effects of LC n-3 PUFA intake on body composition in humans, randomized controlled trials are required. Such trials are few in number; hence there is little information regarding effects of LC n-3 PUFA on body composition when consumed either alone or in conjunction with dietary energy restriction and/or regular exercise.

#### 3.2.1. Effect of LC n-3 PUFA Intake Alone on Body Composition

Couet *et al.* [23] conducted a single-blind intervention trial in which six healthy adults (5 male, 1 female) consumed a control diet (52% carbohydrate, 16% protein, 32% fat; as % of energy intake) for three weeks then, after a 10–12 week washout, consumed the same diet but with 6 g/day of visible fat replaced with fish oil (providing 1.1 g/day EPA and 0.7 g/day DHA). At the beginning and end of each dietary period resting metabolic rate and fat oxidation were measured by indirect calorimetry and body composition was assessed by dual-energy x-ray absorptiometry (DXA). Dietary intakes (7 day recall) and physical activity (by activity recall) were completed two weeks prior to each experimental period to monitor any changes in diet or physical activity. Resting fat oxidation was increased (22%) and body fat was reduced (−0.88 kg) on the fish oil diet; these differences were not accounted for by any differences in dietary intake or physical activity. However, one major limitation of this study was that, rather than balancing the order in which the dietary treatments were undertaken, all volunteers consumed the control diet followed by the fish oil diet and this may have introduced an order effect, potentially confounding the outcome.

Subsequently, Kabir *et al.* [24] studied the effects of supplementation with LC n-3 PUFA on body composition in 27 overweight and obese postmenopausal women with type 2 diabetes. Subjects were randomized in a double-blind parallel study design to consume 3 g/day of EPA-rich fish oil (1.08 g EPA, 0.72 g DHA) or 3 g/day of paraffin oil for two months. Body composition was assessed by DXA and subcutaneous and visceral fat cross-sectional areas were determined by a single–slice computerized tomography (CT) scan at the L4–L5 disc space level. A biopsy of subcutaneous periumbilical adipose tissue was also collected. By the end of the study period, neither treatment group had reduced their body weight and there were no changes in subcutaneous or visceral fat cross–sectional areas assessed by CT. However, the LC n-3 PUFA group reduced their body fat mass significantly (~1.6 kg); this reduction was primarily due to a loss of trunk fat. The authors attributed the discrepancy in findings between the CT scans and DXA as being due to limited sensitivity of a single-slice CT scan for detecting changes in abdominal fat combined with the fact that DXA assessed fat lost from the whole of the trunk and not just the abdomen at the level of the CT slice. Participants randomized to the fish oil treatment also experienced a reduction in adipocyte diameter, whereas this did not change in the control group. The finding of a reduction in adiposity in these postmenopausal women with type 2 diabetes is in contrast to the aforementioned study by Todoric *et al.* [14] in diabetic mice wherein dietary LC n-3 PUFA supplementation exacerbated increases in body fat during high fat feeding. The reason for the discrepancy in findings between these two studies is not clear, but it is possible that effects of LC n-3 PUFA on body composition are regulated differently in mice that are genetically predisposed to diabetes, and humans who develop type 2 diabetes as a result of lifestyle habits. The findings from the study by Kabir *et al.* [24] also refute the possible gender influences on body fat reduction observed in large cohort studies [18,19].

#### 3.2.2. Effect of LC n-3 PUFA Intake Combined with Dietary Energy Restriction on Body Composition

Dietary energy restriction is widely accepted as a cornerstone in the management of obesity and a number of studies have examined whether supplementing the diet with LC n-3 PUFA can enhance the weight loss that can be achieved through dietary restriction.

Fontani *et al.* [25] investigated the effects of LC n-3 PUFA supplementation against a background of body fat reduction using two different controlled diets. Thirty three non-competitive athletes were randomized to consume either a low-carbohydrate Zone diet (40% carbohydrate, 30% protein, 30% fat) or a diet recommended by the Italian National Research Institute for Nutrition and Foods (55% carbohydrate, 15% protein, 30% fat) for 70 days. Half of the volunteers on each dietary pattern were further randomized to consume 4 g/day of fish oil (1.6 g EPA, 0.8 g DHA) or 4 g/day of olive oil in a cross-over design (*i.e.*, consume fish oil for 35 days and then cross over to olive oil, or *vice versa*). Body fat was assessed by skinfolds at baseline, and at the end of each 35 day period. The two diets both reduced body fat by approximately 7%, but there was no additional effect of LC n-3 PUFA supplementation. However this study had serious limitations: The oil supplementation periods may have been too short to see effects on body composition and there may have been a carry-over effect of LC n-3 PUFA accumulation in various tissues confounding the outcome in those volunteers who received LC n-3 PUFA initially. Incorporation of LC n-3 PUFA into tissues (e.g., erythrocyte membranes) has been shown to increase by as much as 150% when provided at a similar dose and timeframe (35 days) to that employed by Fontani *et al.* [26]. Retention of LC n-3 PUFA in these tissues is prolonged, and levels can remain elevated for up to 18 weeks [27]. 

More recently, Krebs *et al.* [28] examined the effect of adding LC n-3 PUFA to a low-fat high–carbohydrate weight loss diet on body weight and body composition in overweight women. Ninety three overweight women completed the study in which they were randomized to the weight loss diet (3.3–3.8 MJ/day; 15% protein, 35% fat, 50% carbohydrate) combined with LC n-3 PUFA (5 g fish oil containing 1.3 g EPA, 2.9 g DHA per day) or placebo (5 g oil containing 2.8 g linoleic acid, 1.4 g oleic acid per day), or to a control condition (no weight loss diet) for five weeks to facilitate acute weight loss. This was followed by a staged reintroduction of energy intake to meet weight maintenance requirements (~10,400 kJ/day) by the end of 12 weeks. Body composition was assessed at baseline and after 12 and 24 weeks by DXA. Body weight did not change in the control group, but fell ~12% in both dietary treatment groups, with no difference in the magnitude of weight loss between LC n-3 PUFA and placebo. Similarly, body fat did not change in the control group but was reduced by a similar amount in both diet groups. Therefore, the findings of this study did not provide any support for greater weight loss or differential changes in body composition when LC n-3 PUFA supplementation is combined with an energy restricted diet in overweight women. 

In the most recent study to examine the effects of LC n-3 PUFA intake on body composition when consumed in conjunction with an energy restricted diet, Thorsdottir *et al.* [29] randomized 278 overweight men and women to one of four isocaloric energy-restricted diets (2500 kJ/day caloric restriction, 50% carbohydrate, 20% protein, 30% fat) for eight weeks. The diets contained no seafood (control), lean fish (150 g cod three times per week, 0.3 g LC n-3 PUFA/day), fatty fish (150 g salmon three times per week, 3.0 g LC n-3 PUFA/day), or fish oil (6 × 1 g capsules yielding 1.5 g LC n-3 PUFA/day). The average weight loss was 6.5 kg for men (~7% body weight) and 4.2 kg for women (~5% body weight). Weight loss and reductions in waist circumference were significantly greater in the groups receiving fish or fish oil compared with controls (~1 kg greater weight loss over the first 4 weeks) but these differences were limited to male volunteers. The finding of additional weight loss not only with the fish oil diet but also with the lean and fatty fish diets was interpreted by the authors as suggesting that weight loss might have been facilitated by fish protein which is rich in the amino acid taurine, since taurine has been shown to decrease body weight in mice [30]. However, the fatty fish and fish oil diets (which provided a higher LC n-3 PUFA intake) tended to provide greater weight loss than the lean fish diet even though there was no significant difference in the weight loss achieved between the three diets. This suggests that LC n-3 PUFA may provide some small additional benefit for weight loss, even when the background intake of LC n-3 PUFA is high as the erythrocyte phospholipid content of LC n-3 PUFA was >9% of total phospholipids in all groups, reflecting a high basal LC n-3 PUFA intake.

The finding of a significantly greater weight loss in male volunteers only in the study by Thorsdottir *et al.* [29] suggests that the lack of any additional weight loss in the study by Krebs *et al.* [28] when LC n-3 PUFA were added to an energy restricted diet may have been because only females were evaluated. However, too few studies have been conducted to date to confirm any gender related difference in the efficacy of LC n-3 PUFA to promote additional improvements in body composition when combined with an energy restricted diet.

#### 3.2.3. Effect of LC n-3 PUFA Intake Combined with Regular Exercise on Body Composition

In addition to energy restriction, increasing physical activity is widely advocated to improve body composition in obesity. As a result, a number of studies have examined whether combining LC n-3 PUFA supplementation with a program of regular exercise provides any additional improvements in body composition beyond what might be achieved through exercise alone.

Warner *et al.* [31] randomized thirty four dyslipidemic adults with ~30% body fat to a control group (usual lifestyle) or one of three treatment groups. For 12 weeks the treatment groups consumed either 50 mL of corn oil per day, 50 mL of fish oil per day (17% EPA, 12% DHA), or consumed 50 mL of fish oil and undertook a progressive aerobic exercise program which consisted of walking or jogging three days per week for 45–50 min at 70–85% of maximal heart rate. Body composition was determined by hydrostatic weighing at baseline and after the 12 week intervention, and dietary intake was assessed by 48 hr dietary recall. The volunteers who undertook the exercise program and consumed fish oil exhibited a 3% reduction in body fat, while body fat was unchanged in the other groups. However, the lack of an exercise control (*i.e.*, no group undertook an exercise only intervention) made it impossible to determine whether this reduction in body fat was due to the exercise alone or to some interaction between the fish oil and exercise.

Brilla and Landerholm subsequently investigated the effects of 10 weeks of fish oil supplementation and exercise on body composition in healthy, previously sedentary young males [32], but included an exercise control group in the study design. Thirty-two healthy young male volunteers were randomized to four groups: control, fish oil, exercise, or fish oil and exercise. The fish oil groups consumed 4 g/day of LC n-3 PUFA. The exercise groups performed aerobic exercise for one hour three times per week. Percent body fat (hydrostatic weighing) and dietary intake (3 day diet records) were assessed at baseline and after 10 weeks. Body fat did not decrease significantly in any treatment group. Unlike previous studies in which LC n-3 PUFA had been shown to reduce body fat in overweight and obese volunteers, the young male volunteers in this study were lean (15–20% body fat) which may have limited the capacity for the LC n-3 PUFA to provide any reduction body fat. Indeed, the use of a lean population may also have accounted for the lack of any reduction in body fat with exercise.

**Figure 1:**
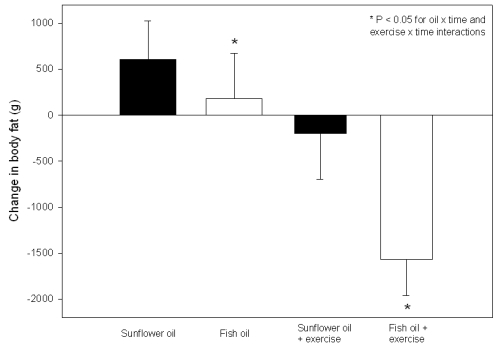
Dual-energy X-ray absorptiometry assessed changes in body fat mass after supplementation with 6 g/day of docosahexaenoic acid-rich fish oil, 6 g/day sunflower oil, fish oil and exercise or sunflower oil and exercise for 12 weeks in overweight and obese adults. * Significant oil × time and exercise × time interactions were detected (*P* < 0.05). Adapted from Hill *et al.* [33] with permission from the American Society for Nutrition.

More recently, Hill *et al.* [33] studied the effects of exercise and fish oil supplementation in overweight and obese adults. Participants were randomized (double-blind) to consume 6 g/day of fish oil containing ~1.9 g LC n-3 PUFA (26% DHA, 6% EPA) or 6 g/day of sunflower oil for 12 weeks. Half of the volunteers in each oil treatment group were further randomized to maintain their usual physical activity or undertake a program of regular exercise which involved walking three times per week for 45 min at 75% of their age-predicted maximum heart rate. Assessment of 3-day weighed food records from 65 subjects who completed the intervention indicated that there were no differences in energy intake between treatment groups. Body weight was reduced by the exercise but there was no effect of LC n-3 PUFA. However, both exercise and LC n-3 PUFA supplementation exerted independent (but additive) effects on body fat reduction; volunteers who undertook exercise and LC n–3 PUFA supplementation lost ~1.6 kg of body fat over 12 weeks (see Figure 1). Without the exercise program, a modest reduction of body fat was achieved by regular supplementation with ~1.9 g/day of LC n-3 PUFA compared with sunflower oil (predominantly n-6 PUFA). Further studies should be undertaken to evaluate the effects of higher doses of LC n-3 PUFA over longer time frames on body fat reduction in overweight and obese populations.

#### 3.2.4. Effect of LC n-3 PUFA Intake Combined with Dietary Energy Restriction and Regular Exercise on Body Composition

While some studies have examined whether combining LC n-3 PUFA with diet or exercise provides any additional benefit for improving body composition, only one study has examined the combination of LC n-3 PUFA with both dietary energy restriction and a program of regular exercise. Kunešová *et al.* [34] reported a 1.5 kg greater weight loss over a 3-week period in severely obese women who undertook a regular exercise program combined with consuming a very low-calorie diet that was supplemented with 2.8 g/day of LC n-3 PUFA (predominantly EPA), compared with those undertaking the exercise program while consuming the very low-calorie diet but receiving a placebo. Twenty seven severely obese women underwent a one week outpatient weight stabilization period, followed by a three week inpatient weight reduction period. During the weight reduction period volunteers undertook 60 min/day of light to moderate physical activity and were randomly assigned to consume either a very low calorie diet (2200 kJ/day) supplemented with 2.8 g/day of LC n-3 PUFA (2:1 ratio of EPA to DHA) or the very low calorie diet supplemented with saline solution. Weight loss was greater in the LC n-3 PUFA supplemented group but did not quite reach statistical significance (7.6 ± 1.8 *vs.* 6.1 ± 2.2 kg, *P* < 0.10), whereas there were significantly greater reductions in body mass index (2.8 ± 0.6 *vs.* 2.2 ± 0.7 kg/m^2^, *P* < 0.05) and hip circumference (4.8 ± 1.8 *vs.* 2.5 ± 2.5 cm, *P* < 0.05) in this treatment group compared with controls. The reduction in body mass index was inversely related to the incorporation of DHA into plasma phospholipids (r = −0.60, *P* < 0.008). While body fat changes were not reported, the authors did find a greater increase in serum beta–hydroxybutyrate in the LC n-3 PUFA supplemented group compared with control, and interpreted this as providing evidence of greater fat oxidation in this treatment group. These findings indicate that the addition of LC n-3 PUFA to a relatively short (3 week) weight loss program comprising severe caloric restriction and exercise may increase fat oxidation leading to greater improvement in body composition.

The finding of this study contrasts with the study by Krebs *et al.* [28] which reported no additional reduction in body weight when LC n-3 PUFA were combined with caloric restriction. The reason for the discrepant findings is not immediately obvious; both studies combined caloric restriction with 3–4 g/day of LC n-3 PUFA to achieve weight loss in overweight and/or obese women. A possible explanation may lie in the magnitude of weight loss achieved, with the study by Krebs *et al.* [28] achieving approximately double the weight loss reported in the study by Kunešová *et al.* (12% of body weight *vs.* 6% of body weight) [34]. It is therefore possible that the much larger weight loss achieved in the study by Krebs *et al.* [28] may have masked any additional weight loss that might have been achieved as a result of LC n-3 PUFA supplementation.

### 3.3. Summary of Effects of LC n-3 PUFA Intake on Body Composition in Humans

In summary, evidence indicates that increasing the intake of LC n-3 PUFA by 0.3–3.0 g/day is effective for reducing body weight and improving body composition in humans. However, these findings come from short-term studies (3–12 weeks in duration) and have for the most part shown that LC n-3 PUFA promote modest reductions in body weight and improvements in body composition when taken either alone, or in conjunction with an energy restricted diet. There is an important need to examine the longer-term effects of LC n-3 PUFA supplementation on body fat in overweight and obese adults and thereby determine if LC n-3 PUFA supplementation may be an effective long-term strategy for body fat reduction.

## 4. Mechanisms of Body Fat Reduction with LC n-3 PUFA

Increasing the dietary intake of LC n-3 PUFA by 0.3–3.0 g/day may assist with body weight and body fat reduction by multiple mechanisms. One mechanism is through an enhancement of post–prandial satiety which might lead to reduced food intake. Another mechanism, and one for which there is the most evidence, is through alterations in the expression of genes involved in the regulation of fat oxidation in adipose, liver, cardiac, intestinal and skeletal muscle tissue, and in the regulation of adipogenesis in adipose tissue. These effects on gene expression favoring enhanced fat oxidation and reduced fat deposition. 

### 4.1. Appetite Effects

Most studies in animals and humans which have shown effects of LC n-3 PUFA on body weight and/or body composition indicate that these effects are independent of energy intake [11,12,17,33]. However, a recent sub-analysis (*n* = 233) [35] of a larger weight loss study (*n* = 278) [29] which showed that weight loss achieved through caloric restriction was enhanced when fish or fish oil was included in the diet, indicated that the intake of LC n-3 PUFA was associated with greater satiety immediately after and 120 min after a test meal. Appetite was assessed during the final two weeks of the eight week dietary intervention in which volunteers had been randomly assigned to one of four isocaloric energy restricted diets (2,100–3,500 kJ/day restriction) consisting of a control diet which contained no seafood, a diet containing lean fish (0.3 g LC n-3 PUFA/day), a diet containing fatty fish (3.0 g LC n-3 PUFA/day), or a diet including six fish oil capsules per day (1.5 g LC n-3 PUFA/day). Weight loss and decrease in waist circumference was significantly greater in the groups receiving fish or fish oil compared with controls (~1 kg greater weight loss over the first four weeks), but in male volunteers only. For the sub-study a validated visual analogue scale was used to assess hunger sensations (fullness, hunger) directly after and two hours after a test meal consisting of 22% protein, 30% fat and 48% carbohydrate. The test meal supplied 35% of the prescribed daily energy intake (1.8–2.9 MJ) and food included in the test meals was representative of the different group specific diets with regard to inclusion of lean or fatty fish and LC n-3 PUFA intake. The visual analogue scale assessment revealed higher sensations of fullness in the volunteers who consumed the higher LC n-3 PUFA content meals (fatty fish and fish oil) compared with those who consumed the lower LC n-3 PUFA content meals (control and lean fish groups) immediately after and two hours after consuming the meal. Feelings of hunger were also lower in the higher LC n-3 PUFA groups two hours after the test meal. The authors concluded that LC n-3 PUFA intake modulates postprandial satiety in overweight and obese volunteers during weight loss and postulated that this might improve compliance with the nutritional treatment of overweight and obesity. Thus, it is possible that increased feelings of satiety following a meal containing a high LC n-3 PUFA content might reduce subsequent food intake, leading to improvements in weight loss and body composition. However, additional research is required. 

### 4.2. Effects on Expression of Genes Regulating Metabolic Pathways

Mitochondrial carnitine palmitoyl transferase I (CPT-I) exchanges coenzyme A for carnitine to facilitate the transfer of acyl groups into the mitochondria for β-oxidation in liver, cardiac muscle and skeletal muscle cells, and is the main control point for β-oxidation. The mitochondrial expression of CPT-I is regulated upstream by peroxisome proliferator-activated receptors (PPARs) [36] and by 5'–AMP-activated protein kinase (AMPK), with the latter having recently been shown to be activated by EPA in both adipose tissue [37] and skeletal muscle [38]. This activation of AMPK by EPA might account for the earlier observation by Power and Newsholme of an increase in CPT-1 with an increased dietary intake of LC n-3 PUFA [39]. Power and Newsholme [39] fed male weanling Lewis rats *ad libitum* for up to 10 weeks on either a low fat diet (25 g corn oil/kg) or one of five high fat diets, comprising 200 g/kg of hydrogenated coconut oil, olive oil, safflower oil, evening primrose oil or EPA rich menhaden (fish) oil. Groups of 12–18 rats were fed each diet and groups of 4–6 animals were removed after 2, 4 and 10 weeks for analysis of cardiac CPT-I and after 4 and 10 weeks for analysis of skeletal muscle CPT-I. Rats fed the EPA-rich menhaden oil had significantly greater CPT-I specific activity in heart and skeletal muscle and significantly greater CPT-1 tissue capacity in skeletal muscle compared with rats fed the low fat diet. While some of the safflower oil and evening primrose oil diets also increased CPT-I specific activity in the heart, only menhaden oil increased mitochondrial CPT-I activity in skeletal muscle. In rats fed menhaden oil, cardiac and skeletal muscle CPT-I had lower sensitivity to inhibition by malonyl CoA compared with rats fed the low fat diet. This lower sensitivity of CPT-I would also be likely to contribute to increased fat oxidation by maintaining a higher CPT-I activity in the face of other dietary effects on CPT-I regulation by malonyl CoA [40]. The increased expression of CPT-I in heart and skeletal muscle as a result of increased LC n-3 PUFA intake is likely to contribute to regulation of the balance between fat and carbohydrate oxidation by promoting an increased transport of fatty acids into the mitochondria and their subsequent oxidation.

Apart from increases in heart and skeletal muscle CPT-I, supplementing the diet of rats with LC n-3 PUFA has also been shown to increase the expression of uncoupling protein 3 (UCP-3) mRNA in skeletal muscle, and the expression of peroxisomal acyl-CoA oxidase in skeletal muscle, liver and heart [9]. Baillie *et al.* [9] fed two groups of 3-4 week old male Fisher 344 rats (*n* = 5 per group) a diet containing 40% of energy from corn oil or fish oil for six weeks, after which time the epididymal (*i.e.*, visceral) fat pads from the rats fed the fish oil diet weighed 25% less than those of the rats fed the corn oil. This decrease in fat deposition was associated with a significant increase in the abundance of skeletal muscle UCP-3 mRNA and an increase in peroxisomal acyl-CoA oxidase gene expression in liver, skeletal muscle and heart. An increase in peroxisomal fatty acid oxidation is important in terms of regulating energy balance because it is a much less efficient pathway for energy production than mitochondrial β-oxidation, yielding 30–40% more heat and 30% less ATP than mitochondrial oxidation [41]. An increase in peroxisomal acyl-CoA oxidase gene expression suggests that LC n-3 PUFA increase the oxidative capacity of this pathway. Additionally, uncoupling proteins reduce the efficiency of mitochondrial oxidative phosphorylation by inducing mitochondrial proton leakage, resulting in a reduced ATP yield from oxidative phosphorylation and an increase in heat production. Thus, the combination of an increase in the expression of uncoupling proteins and peroxisomal acyl–CoA oxidase would be expected to result in a reduction in overall metabolic efficiency, thereby potentially contributing to an increased loss of energy in the form of heat and less storage of energy in the form of fat.

In addition to these studies which have demonstrated alterations in gene expression in skeletal muscle, liver and cardiac muscle, Mori *et al.* [42] examined the effects of supplementing the diet with LC n-3 PUFA on body fat accumulation and the potential role of changes in intestinal gene expression in mediating a reduction in body fat accumulation. Mori and coworkers divided obesity-prone C57BL/6J mice into five treatment groups and fed them a low fat diet (5% triglyceride by weight), or one of four high fat diets (30% by weight) for five months. The high fat diets consisted of 30% triglyceride, or 2%, 4% or 8% fish oil with the balance made up of triglyceride. The animals supplemented with 8% fish oil plus 22% triglyceride exhibited an attenuation (*P* < 0.05) of body weight increase (13.7 g increase) compared with the 30% triglyceride diet (20.8 g increase). The increase in body weight in this treatment group did not differ from that of the mice fed the low-fat (5%) diet (14.6 g). The differences in body weight gain between the 8% fish oil group and the 30% triglyceride group could not be accounted for by differences in energy intake. However, the attenuation of weight gain in the 8% fish oil group was associated with increased intestinal expression of genes coding for CPT 1a, cytochrome P450 4A10 and malic enzyme and 1.2-, 1.6- and 1.7-fold increases in enzymes of β-oxidation, ω-oxidation and malic enzyme respectively compared to those receiving the 30% triglyceride diet. The authors concluded that the anti-obesity effect of fish oil was associated with an up-regulation of intestinal lipid oxidation. 

Therefore, part of the mechanism by which LC n-3 PUFA reduce body fat may be through promotion of fat oxidation and, in particular, the less efficient peroxisomal oxidation of fat combined with less efficient mitochondrial substrate oxidation due to upregulation of uncoupling proteins in liver, cardiac muscle and particularly skeletal muscle which is a major site for whole body lipid metabolism [43], as well as through an increase in intestinal lipid oxidation. Taken together, these effects would be expected to lead to increased but less efficient fat oxidation (and potentially less efficient mitochondrial oxidation of other substrates), contributing to a reduced availability of substrate for uptake and storage of fat by adipose cells.

While these effects on intestinal tissue, skeletal muscle, liver and heart may promote fat oxidation and reduce metabolic efficiency in these tissues, contributing to a reduction in substrate availability for storage by adipose cells, there is also evidence that feeding fish oil influences fat metabolism in adipose tissue itself. Indeed, there is evidence that LC n-3 PUFA results in changes in gene expression in adipose tissue which favor the oxidation of fat and reduce the capacity to synthesize fat in visceral fat depots.

Flachs *et al.* [44] fed 4-month old male C57BL/6J mice for four weeks on two diets containing 20% fat (w/w), with the fat in these diets consisting of either flaxseed oil (rich in α-linolenic acid (ALA, a short chain n-3 PUFA) or flaxseed oil admixed with an LC n-3 PUFA concentrate (6% EPA, 51% DHA). Two further groups of mice were fed for five weeks on two diets containing 35% fat (w/w), one very low in LC n-3 PUFA and the other admixed with LC n-3 PUFA concentrate which replaced 15% of the dietary lipids. Mice on the LC n-3 PUFA supplemented diets had significantly lower body weights and epididymal (*i.e.*, visceral) fat weights by the end of the study periods compared with mice on the non–LC n-3 PUFA supplemented diets but there were no differences in dorsolumbar (*i.e.*, subcutaneous) fat weight. The greater reduction in epididymal fat with the LC n-3 PUFA supplemented diets was associated with increased β-oxidation and decreased fatty acid synthesis in epididymal fat and the upregulation of a number of genes coding for mitochondrial proteins. In particular there was an up-regulation of peroxisome proliferator-activated receptor gamma coactivator 1α (PGC-1α), CPT-I and nuclear respiratory factor-1 (NRF-1) in epididymal fat but not subcutaneous fat. Given that PGC-1α is an upstream regulator of mitochondrial biogenesis and oxidative metabolism, while CPT-I regulates fatty acid entry to the mitochondria and NRF-1 regulates mithochondrial biogenesis [45], these changes in gene expression would be expected to increase the capacity of epididymal fat to oxidize fatty acids. This was observed as an increase in β-oxidation in this tissue, as assessed by the release of radioactivity into tissue culture medium from fragments of adipose tissue prelabelled with [9,10(n)-^3^H]oleic acid. The diets admixed with LC n-3 PUFA also resulted in a reduction in expression of stearoyl-CoA desaturase mRNA (a key enzyme in the lipogenic pathway) in epididymal fat, and this was associated with a reduction in fatty acid synthesis in this tissue as assessed by ^3^H_2_O incorporation into saponifiable fatty acids. Thus, taken together, the findings of this study by Flachs *et al.* [44] indicate that LC n-3 PUFA increase the expression of genes in epididymal (*i.e.*, visceral) fat which favor fatty acid oxidation and reduce fatty acid synthesis.

Overall, an increased oxidation of fat in heart, liver, skeletal muscle and visceral fat would lead to a reduction in availability of substrate for deposition in adipose tissue, while a reduced capacity to store fat as a result of reduced expression of lipogenic genes in this tissue would contribute to an overall reduction in body fat. While the aforementioned alterations in gene expression would favor a reduction in body fat, many of these changes are not necessarily specific for individual LC n-3 PUFA. A recent preliminary study using microarray analysis of genes regulated by dietary fatty acids has shown considerable overlap in the influence of different PUFA on PPARs and other nuclear receptors [46]. Therefore, the net influence on fat metabolism will be determined by a complex integration of the effects of individual PUFA on gene expression.

### 4.3. Effects on Tissue Anabolism and Maintenance of Lean Tissue Mass

There is some evidence from a limited number of animal studies which suggests that LC n-3 PUFA may alter the activity of anti-catabolic and/or anabolic pathways in skeletal muscle to promote the maintenance of lean tissue mass. Any such promotion of lean tissue mass would be expected to increase metabolic rate, and might therefore contribute indirectly to reductions in body fat. EPA has been shown to suppress activation of the ubiquitin-proteasome pathway [47], which is the key pathway responsible for muscle proteolysis during energy restriction [48], by impairing activation of the transcription factor NF-κβ, an important regulator of this pathway [47,49]. An increased intake of LC n-3 PUFA has also been shown to increase whole body protein synthesis via the activation of key regulatory kinases involved in muscle protein synthesis, specifically mTOR and S6K in steers [50]. These potential anti-catabolic and anabolic effects of LC n-3 PUFA may attenuate muscle loss and promote the maintenance of muscle mass (and metabolic rate) during periods of dietary energy restriction, or potentially increase muscle mass during energy balance. Additional studies of the potential for LC n-3 PUFA to maintain lean tissue mass during energy restriction appear warranted, particularly in humans.

### 4.4. Effects on Muscle Blood Flow

Arterial vasodilator function [51,52] and skeletal muscle blood flow [53] are impaired in obesity and are associated with reduced nutrient disposal [54]. We recently demonstrated that supplementation with 1.9 g/day of LC n-3 PUFA improved vasodilator function [33] while Walser *et al.* [55] showed that increased LC n-3 PUFA (5 g/day) increased skeletal muscle blood flow during exercise. Thus, some of the observed reductions in body fat with LC n-3 PUFA may be in part due to improved vasodilator function increasing blood flow and nutrient delivery to skeletal muscle resulting in improved nutrient utilization for energy production and less conversion to fat storage. No studies have directly addressed this hypothesis, but given the inter-relationships between obesity, skeletal muscle blood flow and nutrient disposal, additional studies seem warranted.

## 5. Conclusions

There is a considerable body of evidence from rodent studies indicating that supplementing the diet with LC n-3 PUFA can attenuate weight gain and reduce body fat, in particular epididymal (visceral) fat. Similarly, in human studies there is a growing body of evidence indicating that increasing the intake of LC n-3 PUFA by 0.3–3.0 g/day can reduce body weight and body fat in overweight and obese individuals.

The mechanism by which increasing the intake of LC n-3 PUFA by such an amount (*i.e.*, 0.3–3.0 g/day) may improve body composition is most likely altered gene expression favoring increased fat oxidation in adipose, liver, cardiac, intestinal and skeletal muscle tissue and reduced fat deposition in adipose tissue. There is some preliminary evidence that an increase in LC n-3 PUFA intake within this dose range might also attenuate postprandial sensations of hunger, although the majority of studies, including those in rodents, which have reported improved body composition, have not reported any reduction in food (*i.e.*, energy) intake. Data from a number of studies also suggest that LC n-3 PUFA might promote increases in lean tissue mass, thus potentially increasing metabolic rate and indirectly assisting with body fat reduction. It is also possible that the increases in vasodilator function and muscle blood flow during exercise that result from supplementation with 1.9–5.0 g/day of LC n-3 PUFA might promote nutrient disposal by skeletal muscle, thus reducing the availability of nutrients for lipogenesis and storage in adipose tissue.

While there is growing evidence that LC n-3 PUFA can improve body composition in humans, there is still much controversy because the majority of studies have been of relatively short duration and the magnitude of the improvements have been modest. Accordingly, there is an urgent need for longer-term studies to determine the relative effects of EPA and DHA supplementation on body composition and the feasibility of using LC n-3 PUFA supplementation as a strategy to improve body composition in overweight and obese populations. 
